# Intracellular cholesterol trafficking and its impact on inflammatory processes

**DOI:** 10.3389/fimmu.2026.1923810

**Published:** 2026-10-30

**Authors:** Nicola Böffinger, Michelle Cheuk-Chi Weidmann, Sofie Patrizia Meyer, Bernhard Brüne, Tobias Schmid

**Affiliations:** 1 Institute of Biochemistry I, Faculty of Medicine, Goethe University Frankfurt, Frankfurt, Germany; 2 German Cancer Consortium (DKTK), Partner Site Frankfurt, Frankfurt, Germany; 3 Frankfurt Cancer Institute, Goethe University Frankfurt, Frankfurt, Germany

**Keywords:** cholesterol, inflammation, intracellular trafficking, lipid transfer protein (LTP), membrane contact site (MCS), NLRP3 inflammasome, pattern recognition receptor (PRR), toll-like receptor (TLR)

## Abstract

Inflammation is a fundamental physiological response to infection and cellular damage, initiated by pathogen- and damage-associated molecular patterns (PAMPs and DAMPs) through pattern recognition receptors located at the plasma membrane and within intracellular compartments. Increasing evidence highlights that inflammatory signaling is closely linked to membrane cholesterol and its intracellular distribution and trafficking. Cholesterol homeostasis is maintained through tightly controlled biosynthesis, uptake, and efflux pathways, and is further shaped by complex intracellular trafficking routes involving vesicular transport and non-vesicular transfer at membrane contact sites. In particular, cholesterol-rich membrane domains, i.e. lipid rafts, have been implicated in toll-like receptor (TLR) signaling, while an activation of this signaling pathway in turn can lead to increased cellular cholesterol levels. Furthermore, cholesterol biosynthesis, intracellular cholesterol trafficking as well as accumulation can promote activation of the nod-like receptor family pyrin domain-containing 3 (NLRP3) inflammasome. This review summarizes the current understanding of cholesterol sensing, distribution, and trafficking pathways and discusses how these processes regulate innate immune signaling. We further highlight the role of cholesterol-mediated modulation of TLR4 signaling and NLRP3 inflammasome activation and explore implications for diseases.

## 1 Introduction

In mammalian cells cholesterol accounts for 20-30% of total cellular lipids. It is essential as a major structural component of cellular membranes in all cells and is involved in regulating lipid metabolism, signaling pathways, and immune function. A tightly controlled balance between synthesis, uptake, efflux, and cellular distribution is therefore required for its physiological role (1). In this mini review, we discuss how cholesterol distribution and trafficking affect inflammatory processes.

## 2 Cholesterol sensing and distribution

The plasma membrane (PM) contains up to 90% of the intracellular cholesterol content, cholesterol accounting for 30–40 mol% of PM lipids (2, 3). PM cholesterol can be divided into three pools: the essential pool, sequestered by phospholipids and membrane proteins, the sphingomyelin (SM)-sequestered pool, and the accessible cholesterol pool. While the latter varies depending on cellular cholesterol availability, the sequestered pools are kept rather stable (3). Interestingly, only 1% of total cellular cholesterol is found at the site where its levels are actually sensed, i.e. the endoplasmic reticulum (ER) (4).

### 2.1 Cholesterol homeostasis

#### 2.1.1 Cholesterol biosynthesis

Cells obtain cholesterol mainly through *de novo* synthesis, which predominantly occurs in the ER and in part in the cytoplasm and peroxisomes (5–7). It begins with acetyl-Coenzyme A (CoA) and involves many enzymatic steps, including the rate-limiting conversion of 3-hydroxy-3-methylglutaryl (HMG)-CoA to mevalonate by the HMG-CoA reductase (8, 9) (**Figure 1A**). As cholesterol synthesis is rather energy-intensive, requiring oxygen, adenosine triphosphate (ATP), and the reduction equivalents reduced nicotinamide adenine dinucleotide (NADH) and NAD phosphate (NADPH), it demands tight regulation by the sterol regulatory element-binding protein 2 (SREBP2)/SREBP cleavage-activating protein (SCAP)/insulin-induced gene 1 (INSIG1) sensory and regulatory system (10). In detail, under high cholesterol conditions, the SREBP2/SCAP complex is retained within the ER membrane by INSIG1. Specifically, abundant sterols bind to SCAP, thereby inducing a conformational shift that disturbs the interaction between SCAP and the proteins responsible for its incorporation into coat protein (COP) II-coated vesicles, and instead increases the affinity between SCAP and INSIG1 (11–13). Furthermore, oxysterols bind directly to INSIG1, which enhances its interaction with SCAP and further hinders SCAP binding to COPII vesicles (14, 15). However, when ER membrane cholesterol levels fall below 5 mol%, SCAP/SREBP2 complexes are released from INSIG1, and shuttled to the Golgi network via COPII vesicles (4). Golgi-resident site-1 protease (S1P) and S2P successively process SREBP2 into an active transcription factor, which translocates to the nucleus and activates the expression of essentially all genes responsible for cholesterol biosynthesis and uptake (16, 17).

**Figure 1:**
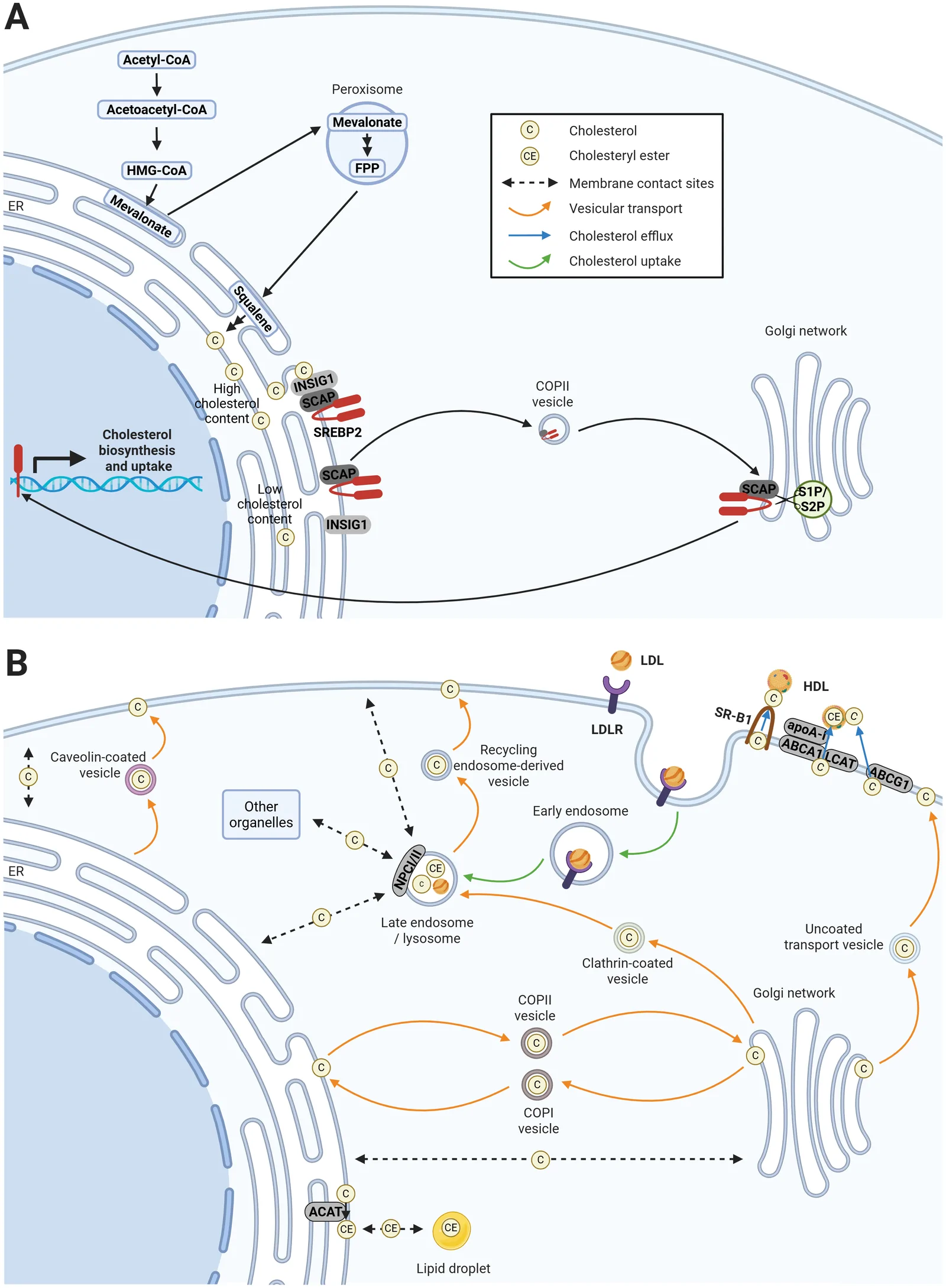
Cholesterol synthesis, sensing, and intracellular distribution. **(A)** Cholesterol synthesis starts in the cytoplasm with the conversion of acetyl-Coenzyme A (CoA) to acetoacetyl-CoA and 3-hydroxy-3-methylglutaryl (HMG)-CoA. Upon import into the endoplasmic reticulum (ER), HMG-CoA is converted into mevalonate, which is further processed in the peroxisomes to farnesyl pyrophosphate (FPP). In the ER, two FPP molecules condensate to squalene, which is further processed to cholesterol (C). Cholesterol levels are sensed in the ER by the sterol regulatory element-binding protein 2 (SREBP2)/SREBP cleavage-activating protein (SCAP)/insulin-induced gene 1 (INSIG) complex. Under low cholesterol conditions, SREBP2/SCAP are released from INSIG1 and transported via coat protein II (COPII) vesicles to the Golgi network. Here, SREBP2 undergoes cleavage-dependent activation by the site-1 protease (S1P) and S2P. The N-terminal part of SREBP2 translocates into the nucleus and acts as a transcription factor activating gene expression involved in cholesterol biosynthesis and uptake. **(B)** Cells take up extracellular cholesterol via low density lipoprotein (LDL) binding to the LDL receptor (LDLR) and subsequent endocytosis (green arrows). Cholesteryl esters (CE), originating from LDL, are processed in late endosomes/lysosomes to cholesterol, which is distributed intracellularly via vesicular (orange arrows) and non-vesicular transport at membrane contact sites (black, dashed arrows). Non-vesicular cholesterol transport from late endosomes/lysosomes involves Niemann-Pick complex I and II (NPCI/II) proteins and facilitates cholesterol distribution to the plasma membrane (PM), the ER, and other organelles. From the ER, membrane contact sites allow for the transport to PM and Golgi network. Vesicular transport includes the interchange of cholesterol between the Golgi network and the ER by coat protein (COP) vesicles. From the Golgi network cholesterol is additionally transported in uncoated transport vesicles to the PM or by clathrin-coated vesicles to the late endosomes/lysosomes. From there, recycling endosome-derived vesicles can transport cholesterol to the PM. Caveolin-coated vesicles establish a direct vesicular transporting route between the ER and the PM. Excess cholesterol is converted into cholesteryl esters in the ER by acyl coenzyme A:cholesterol acyltransferase (ACAT) and subsequently stored in lipid droplets in the cytoplasm. Export of cholesterol (blue arrows) and corporation into high density lipoprotein (HDL) is mediated by scavenger receptor type B 1 (SR-B1), or by the interplay between ATP-binding cassette (ABC) transporters ABCA1, apolipoprotein A-I (apoA-I), and lecithin-cholesterol acyltransferase (LCAT), converting cholesterol into cholesteryl esters, which are stored inside the HDL. ABCG1 is able to transfer cholesterol from the PM directly onto HDL.

#### 2.1.2 Cholesterol import

In addition, cells can obtain exogenous cholesterol through endocytic uptake. In particular, low density lipoprotein (LDL) particles carrying esterified cholesterol bind to the LDL receptor (LDLR) and are subsequently internalized via clathrin-mediated endocytosis (18, 19) (**Figure 1B**). Following fusion of endosomes and lysosomes, cholesteryl esters are hydrolyzed by lysosomal acid lipase, resulting in the release of free cholesterol from the endolysosome and intracellular distribution (20, 21).

#### 2.1.3 Cholesterol storage and efflux

Since excess intracellular cholesterol accumulation is directly linked to cellular toxicity and the activation of inflammatory pathways, cholesterol storage in lipid droplets and cholesterol efflux is crucial for maintaining cellular homeostasis (22–24). Excess free cholesterol undergoes re-esterification by acyl coenzyme A:cholesterol acyltransferase (ACAT) in the ER and cholesteryl esters are then stored in the hydrophobic cores of lipid droplets as lipid reservoirs (25). Although passive cholesterol efflux, such as passive diffusion or diffusion facilitated by scavenger receptor type B 1 (SR-B1), is possible, the majority of cholesterol efflux occurs via active pathways (26) (**Figure 1B**). Active cholesterol efflux is mediated by the membrane-associated ATP-binding cassette (ABC) transporters ABCA1 and ABCG1 (27, 28). Specifically, ATP hydrolysis leads to the transport of free cholesterol and phospholipids to the extracellular space onto the lipid-poor acceptor apolipoprotein A-I (apoA-I) by ABCA1, resulting in the formation of discoidal nascent high-density lipoprotein (HDL) (26, 29). ApoA-I activates the plasma-resident enzyme lecithin-cholesterol acyltransferase (LCAT), which facilitates extracellular esterification of free cholesterol, thereby promoting the formation of spherical mature HDL (30, 31). After nascent HDL matures, ABCG1, which is unable to directly load apoA-I with free cholesterol, further enhances cholesterol efflux by transferring cholesterol to mature HDL particles (27).

### 2.2 Intracellular cholesterol distribution

In addition to synthesis, uptake, storage, and efflux, intracellular cholesterol dynamics are further regulated through cholesterol distribution, which occurs via vesicular or non-vesicular transport.

#### 2.2.1 Vesicular cholesterol transport

Although non-vesicular transport accounts for the majority of cholesterol trafficking mechanisms, vesicular trafficking also contributes to sterol redistribution. Protein transport between the ER and the Golgi network is carried out by COPI- and COPII-coated vesicles (**Figure 1B**). Specifically, COPII vesicles are responsible for anterograde transport from the ER to the Golgi network, whereas COPI vesicles mediate retrograde transport from the Golgi network to the ER, and between different Golgi cisternae (32, 33). In addition to their role in protein trafficking, COPI and II vesicles maintain the organelle-specific cholesterol distribution along the secretory pathway, where cholesterol concentrations increase from the ER to the Golgi network to the PM (34). Importantly, how exactly increasing cholesterol concentrations along the secretory pathways are achieved is largely unknown. Since cholesterol concentrations in COPI vesicles are generally lower than within the Golgi network, partial exclusion of cholesterol from COPI vesicles might contribute to the early part of the cholesterol gradient. Cholesterol distribution has further been associated with caveolae-dependent transport pathways. Caveolae are small invaginations located in the PM that are rich in free cholesterol and sphingomyelin (35, 36). Caveolin-1, the main structural protein of caveolae, is synthesized and oligomerized in the ER, and transported to the Golgi network via COPII vesicles. As the membranes of COPII vesicles are enriched with cholesterol, caveolin-1 has been implicated in the transport of cholesterol along the secretory pathway (37, 38). Along similar lines, caveolin-coated vesicles transport newly synthesized cholesterol from the ER to the PM in a COPII-independent manner (39). A minor part of cholesterol further reaches the PM directly from the Golgi network via uncoated transport vesicles or via clathrin-coated vesicles from the endosomal compartment (38, 40).

#### 2.2.2 Non-vesicular cholesterol transport

Non-vesicular cholesterol transport is facilitated by lipid-transporting proteins (LTPs) at membrane contact sites (MCSs) (41, 42). Here, cell organelles come into close proximity allowing for the interchange of proteins, lipids, and other signaling molecules involved in cellular communication and homeostasis (43) (**Figure 1B**).

##### 2.2.2.1 Niemann-Pick complex I and II

Among the most important LTPs are the Niemann-Pick complex (NPC) I and II proteins, which support the transfer of cholesterol from lysosomes and endosomes to other membrane structures (44, 45). In detail, cholesterol binds to the soluble NPCII and is transferred to membrane-embedded NPCI, in turn mediating cholesterol transport through vesicular membranes (46–48). Of note, NPCI interacts with numerous other LTPs to facilitate cholesterol transport at MCSs (49, 50).

##### 2.2.2.2 Oxysterol-binding protein-related proteins

Another essential class of LTPs is the oxysterol-binding protein (OSBP)-related protein (ORP) family (51). OSBP was one of the first discovered cholesterol-binding and -transporting proteins (52). To date, 12 additional ORPs have been identified in mammals, all of which share a C-terminal sterol- or lipid-binding site, the OSBP-related domain (ORD) (53, 54). In addition to OSBP, ORP9, ORP10, and ORP11 are involved in the transport of cholesterol between the ER and the trans-Golgi network at MCSs (55). Notably, because of the different cholesterol levels of the ER and Golgi network, cholesterol transport must occur against a concentration gradient. Therefore, OSBP mediates counter-transport and subsequent hydrolysis of phosphatidylinositol-4-phosphate to provide energy (56). In contrast, ORP1L and ORP5 are involved in cholesterol transport from the late endosomal and lysosomal compartments to the ER, ORP5 acting downstream of NPCI (49, 57). ORP2 facilitates cholesterol transport from ER to the PM in exchange for phosphatidylinositol-4,5-bisphosphate, while the cholesterol transport from the PM to ER is mediated by ORP5 and ORP8 (58, 59).

##### 2.2.2.3 Steroidogenic acute regulatory protein-related lipid transfer-related domain proteins

The steroidogenic acute regulatory protein-related lipid transfer (START)-related domain (STARD) protein family with 15 members represents another class of LTPs, which bind cholesterol via their START domain (51, 60). STARD3 and STARD3 N-terminal like (STARD3NL) facilitate reverse cholesterol transport from the late endosomes to the ER, whereas STARD1 and 3 promote cholesterol transport from the ER (STARD1), or from the late endosomes (STARD3) to mitochondria (61–64). Although STARD4 was identified as a major LTP, it has not yet been attributed to a specific MCS and might play a role in various cholesterol trafficking routes, for example between the PM and the ER or the endosomal recycling compartment (65–67).

##### 2.2.2.4 Aster proteins

Another protein family important for cholesterol transport between the ER and the PM comprises the Aster-A, B, and C proteins, also referred to as glucosyltransferase, rab-like guanosine triphosphatase activators and myotubularins (GRAM) domain-containing proteins (GRAMD)1a, b, and c, respectively (68, 69). These LTPs consist of a transmembrane domain, which anchors them to the ER, a START-like ASTER domain for cholesterol transport, and a GRAM domain for sensing phosphatidylserine and cholesterol in the PM (68, 70). Aster-B assists in sensing total cellular cholesterol contents by detecting the accessible cholesterol pool. It further transports cholesterol from the PM to the ER, thereby contributing to the maintenance of cholesterol homeostasis (71–73). The exact mechanism of this communication between the PM and ER via Aster-B is discussed in depth elsewhere (74). More recently, Aster proteins have also been suggested to control cholesterol levels in the Golgi network by reverse cholesterol transport from the Golgi network to the ER at MCSs (55).

## 3 Cholesterol and inflammation

Cholesterol transport and distribution are tightly connected to inflammatory processes. Pattern recognition receptors (PRRs) cluster in lipid rafts on the PM (75), which are not only strongly enriched in cholesterol, but also contain elevated levels of sphingomyelin and glycophospholipids (76). They have been shown to be involved in receptor- and caveolin-mediated endocytosis and, consequently, are also located in early endosomes. While lipid rafts are not strictly defined due to their dynamic and compositionally variable nature, they are widely accepted as functional membrane microdomains that serve as platforms for PRRs and contribute to inflammatory signaling cascades (77, 78). Furthermore, cholesterol transport itself can affect the signaling of intracellular PRRs, which will be discussed in the following sections.

### 3.1 Cholesterol and toll-like receptor signaling

Toll-like receptors (TLRs) are an important class of PRRs that accumulate in lipid rafts upon activation (79). The TLR family comprises 10 members localized either to the PM or intracellular compartments (80). TLRs recognize pathogen-associated molecular patterns (PAMPs), such as bacterial or viral components, and/or damage-associated molecular patterns (DAMPs) like oxidized LDL particles, and initiate immune responses upon activation by distinct downstream signaling cascades (81, 82).

Within the TLR family, TLR4 represents one of the best characterized receptors. After synthesis and chaperone-mediated folding in the ER, mature TLR4 is transported to the Golgi network via COPII vesicles (83–85) (**Figure 2A**). Thereafter, two distinct pools of TLR4 in the PM and in the endosomal recycling compartment are established, which form an interconnected network of TLR4 trafficking (86). Upon ligand binding on the cell surface, TLR4 dimerizes within lipid rafts and initiates a signaling cascade via the adaptor protein myeloid differentiation primary response gene 88 (MyD88) (87). Ultimately, downstream signaling activates the transcription factor nuclear factor kappa-light-chain-enhancer of activated b cells (NFκB), which enters the nucleus and enhances the expression of pro-inflammatory cytokines (82). Notably, the TLR4/MyD88/NFκB signaling axis promotes the expression of SREBP2 and LDLR, thereby enhancing both cholesterol synthesis and uptake (88, 89). Interestingly, in response to PRR stimulation, new membrane structures are built to cope with damage or infection, for example, to enable recycling of damaged organelles or shuttling of pathogens via the endolysosomal compartment (90, 91). Therefore, increased intracellular cholesterol levels upon TLR4 activation likely directly contribute to inflammatory defense mechanisms. On the other hand, rising intracellular cholesterol levels, either in the PM or in the endosomal compartment, enhance TLR4 signaling, driving a positive feedback loop between high cholesterol levels and TLR4 signaling (92–94). The second axis of TLR4 activation takes place in early endosomes. Binding of intracellular ligands results in the recruitment of the adaptor toll/interleukin-1 receptor domain-containing adaptor protein inducing interferon β (TRIF), which eventually promotes interferon regulatory factor 3 (IRF3)-dependent expression of type I interferons (95). Interestingly, IRF3 downregulates ABCA1, thus, endosomal TLR4 signaling again increases intracellular cholesterol levels (96).

**Figure 2:**
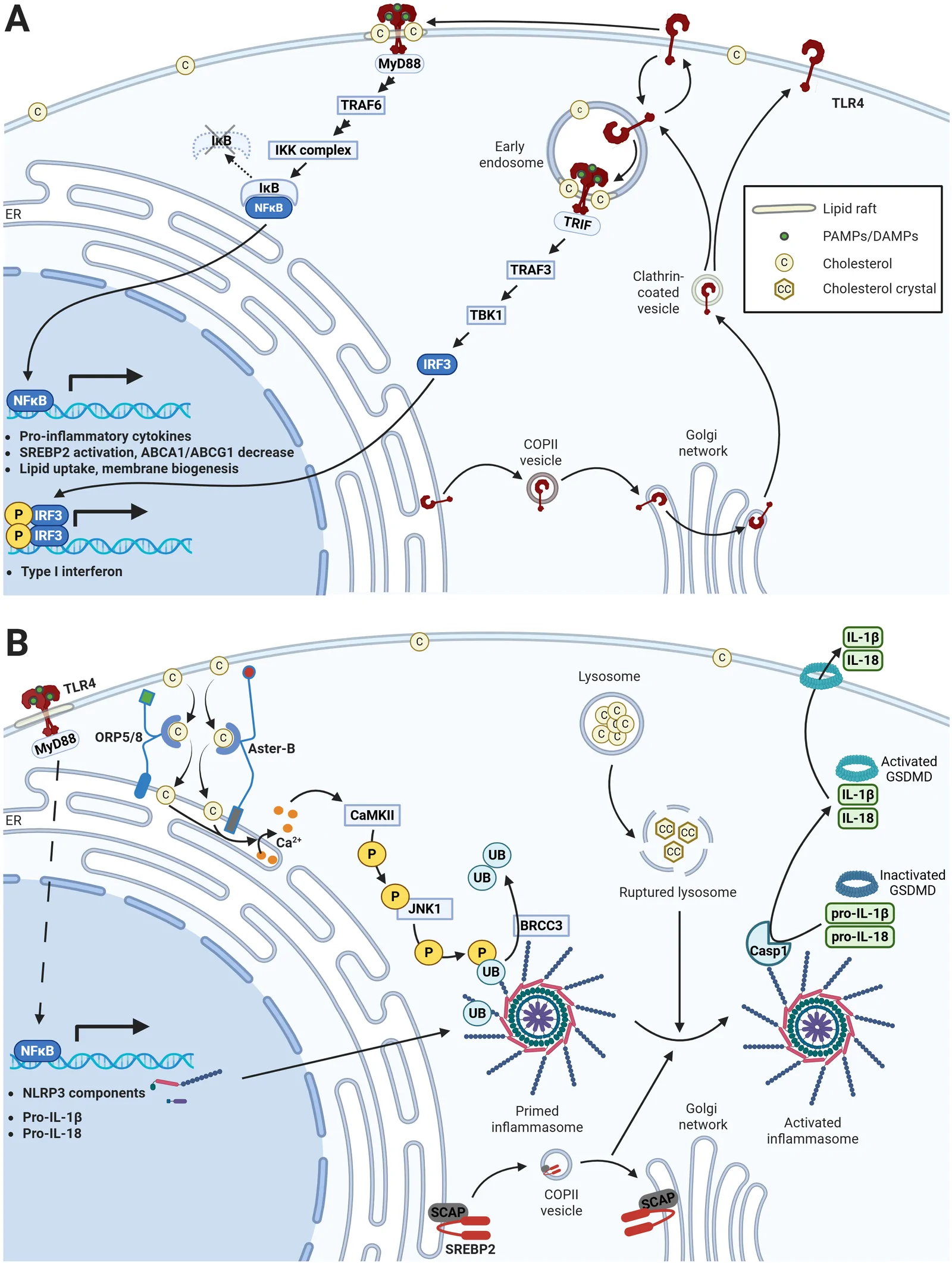
Effect of cholesterol on inflammatory signaling processes. **(A)** Toll-like receptor 4 (TLR4) is synthesized in the endoplasmic reticulum (ER), transported via coat protein II (COPII) vesicles to the Golgi network and distributed to early endosomes and the plasma membrane (PM) via clathrin-coated vesicles. TLR4 can be interchanged between the PM and the early endosomes, and initiates two different signaling pathways upon activation. With the binding of pathogen-associated molecular patterns (PAMPs) or damage-associated molecular pattern (DAMPs), TLR4 dimerizes within lipid rafts. From the PM, the signaling cascade involves myeloid differentiation primary response gene 88 (MyD88), and the E3 ubiquitin ligase tumor necrosis factor receptor-associated factor 6 (TRAF6), which leads to the activation of the inhibitor of kappa-light-chain-enhancer of activated B cells (IκB)-kinase (IKK) complex. The complex phosphorylates the nuclear factor κB (NFκB)-associated IκB to induce its degradation, allowing for the translocation of the transcription factor NFκB into the nucleus, which enhances the expression of pro-inflammatory cytokines and leads to activation of sterol regulatory element-binding protein 2 (SREBP2), suppression of cholesterol exporters ATP-binding cassette (ABC) ABCA1 and ABCG1, and expression of proteins important for lipid uptake and membrane biogenesis. In contrast, activation of endosomal TLR4 activates signaling by the adaptor protein toll/interleukin-1 receptor domain-containing adaptor protein inducing interferon β (TRIF), which recruits TRAF3 and the tumor necrosis factor receptor-associated NFκB activator binding kinase 1 (TBK1). TBK1 activates interferon regulatory factor 3 (IRF3) by phosphorylation, which dimerizes and translocates to the nucleus, enhancing the expression of type I interferons. **(B)** TLR4-dependent MyD88-mediated activation of NFκB further enhances expression of nod-like receptor family pyrin domain-containing 3 (NLRP3) components, pro-interleukin-1β (pro-IL-1β), and pro-IL-18. Upon assembly of the NLRP3 components the primed inflammasome is formed. Another priming and activation signal is induced by the non-vesicular transport of cholesterol (C) from the PM to the ER by Aster-B and oxysterol-binding protein-related protein 5 and 8 (ORP5/8). Elevated cholesterol levels within the ER trigger the release of calcium from the latter, which activates calcium/calmodulin-dependent kinase II (CaMKII), and subsequently c-Jun N-terminal protein kinase 1 (JNK1). JNK1 phosphorylates the ubiquitylated inflammasome, thereby facilitating breast cancer gene A1/A2-containing complex 3 (BRCC3)-mediated de-ubiquitylation, and consequently inflammasome activation. Further cholesterol-mediated activating signals include lysosomal rupture induced by an accumulation of cholesterol crystals (CC) as well as SREBP cleavage-activating protein (SCAP) translocation from the ER to the Golgi network. The activated inflammasome associates with Caspase-1 (Casp1). Casp1 cleaves pro-IL-1β and pro-IL-18 and activates gasdermin D (GSDMD), thus leading to the secretion of the pro-inflammatory cytokines IL-1β and IL-18 through GSDMD-formed pores.

### 3.2 Cholesterol and the nod-like receptor family pyrin domain-containing 3 inflammasome

Nod-like receptors (NLRs) comprise another class of intracellular PRRs, which assemble to form different inflammasomes (97). Inflammasomes are multicomponent protein complexes, which, upon activation, trigger an inflammatory cascade (98). Here, we focus on the NLR family pyrin domain-containing 3 (NLRP3) inflammasome, since its connection to cholesterol is rather well characterized (99). The activity of the NLRP3 inflammasome is tightly regulated at various levels to prevent spontaneous inflammatory signaling (100, 101). Indeed, two signals are necessary to facilitate NLRP3 inflammasome activity, namely priming and activation (102). Priming occurs due to enhanced expression of inflammasome components, or post-transcriptional events (e.g., (de)phosphorylation or deubiquitylation) that allow for inflammasome assembly (103, 104) (**Figure 2B**). Following assembly, the inflammasome is activated by the catalytic cleavage of pro-caspase 1 to the active caspase 1 (Casp1), which is anchored to the inflammasome. Casp1 in turn facilitates cleavage and activation of gasdermin D (GSDMD), which initiates pyroptosis by forming pores in the PM (102). Furthermore, Casp1 enables the processing of pro-inflammatory cytokine precursors pro-interleukin-1β (pro-IL-1β) and pro-IL-18 to their active forms IL-1β and IL-18. Subsequently, the active cytokines are released from the cell via the GSDMD-induced pores and stimulate an inflammatory response (105).

Changes in cholesterol homeostasis or trafficking affect inflammasome signaling in various ways (**Figure 2B**): TLR4 activation induces inflammasome priming via MyD88/NFκB-mediated enhanced expression of NLRP3 components, as well as of pro-IL-1β and pro-IL-18 (106). As high cholesterol levels promote the activation of TLR4 signaling, priming of the inflammasome can be achieved by elevated cholesterol contents. Moreover, the activation of the primed inflammasome is supported by non-vesicular cholesterol transport from the PM to ER (107). Specifically, Aster-B as well as ORP5 and 8 mediate cholesterol transport to the ER to enable sensing of cellular cholesterol levels, and consequently regulation of cholesterol homeostasis (58). Elevated cholesterol levels in the PM enhance LTP-dependent reverse cholesterol transport. The resulting higher ER cholesterol levels promote protein kinase A (PKA)-mediated Ca^2+^ release from the ER, which activates calcium/calmodulin-dependent kinase II (CaMKII) and subsequently activates c-Jun N-terminal protein kinase 1 (JNK1), resulting in phosphorylation of the ubiquitylated inflammasome (107, 108). Phosphorylation then allows for deubiquitylation by the breast cancer gene A1/A2-containing complex 3 (BRCC3), which ultimately operates as an activation signal of the inflammasome (109, 110). Furthermore, lysosomes are involved in inflammasome activation upon high levels of cholesterol: high intracellular concentrations of cholesterol lead to the accumulation of cholesterol crystals in lysosomes, which can also be taken up by cells as pre-formed cholesterol crystals via endocytosis, ultimately accumulating in lysosomes (111). Cholesterol crystals are able to physically trigger lysosome rupture and activate the inflammasome in concert with the released lysosomal enzymes in various cell types (23, 111). Notably, inhibition of NPCI causes cholesterol accumulation in lysosomes without forming crystals and lysosomal rupture. Surprisingly, lysosomal cholesterol accumulation and concurrent decreased cholesterol pool in the ER also activate the inflammasome (112). In line, the process of translocation of SCAP from ER to Golgi network upon low cellular cholesterol levels was found to activate the inflammasome, which contradicts earlier findings and underlines the mutual influence of intracellular cholesterol trafficking and inflammation (113).

## 4 Implications of cholesterol dynamics for diseases

As changes in intracellular cholesterol levels as well as localization and trafficking can trigger inflammatory pathways, their maintenance appears to be crucial for cellular health and disease prevention. This is further underlined by the fact that cholesterol trafficking interacts with many other important pathways. For example, cholesterol transport by Aster proteins regulates hedgehog signaling (114). Furthermore, a link between mechanistic target of rapamycin 1 (mTORC1) and cholesterol transport at ER-lysosomal MCSs has been described (115–117). Thus, it is not surprising, that disturbed cholesterol distribution, homeostasis, and trafficking all are associated with numerous diseases, such as cardiovascular disorders (CVD), cancer, neurodegenerative diseases, and viral infections (118–121). Interestingly, recent research has shown that TLR4 signaling downregulates several ORP family members. This corroborates the crosstalk between cholesterol trafficking and immune signaling, and suggests intracellular cholesterol trafficking as a therapeutic target in inflammatory diseases (122, 123). Despite substantial evidence implicating ORP/OSBP, STARD3, and Aster proteins in inflammatory signaling, direct approaches to pharmacologically target these cholesterol-transfer proteins in inflammatory diseases are rather scarce. However, LTPs have emerged as therapeutic targets in cancer and neurological diseases. For example, increased STARD1 expression was linked to Alzheimer’s disease and Down syndrome, while elevated STARD3 expression was associated with breast cancer (124, 125). Specifically, in Alzheimer’s disease, neuroinflammation is an early pathology-associated response which contributes to disease progression, while elevated mitochondrial cholesterol levels could be linked to increased neuroinflammation. Because STARD1 is acting at the ER-mitochondrial MCS and was found overexpressed in patient’s brains, it emerged as novel diagnostic marker but also as a therapeutic target to prevent disease progression (126, 127). Furthermore, ORP5 and ORP8, as well as Aster expression promotes the progression of different cancer types (128, 129). Aster-A overexpression has been shown to enhance tumor growth and resistance to chemotherapy in hepatocellular carcinoma via signal transducer and activator of transcription 5 (STAT5), which drives the expression of inflammatory factors (130–132). ORP5 was commonly found overexpressed in renal cell carcinoma and associated with tumor progression (133). Moreover it increased cell proliferation via mTORC1 in cervical carcinoma cell lines, while ORP8 overexpression inhibited cell proliferation and decreased tumor growth in gastric tumor models (128, 134). Similarly, OSBP has been associated with viral infections, where cholesterol is often used as an anchor for viral cell entry, further highlighting LTPs as promising targets for therapeutic interventions (135–138). Of note, measures to lower cellular cholesterol concentrations, such as the use of statins (139), enhancing cholesterol efflux by activating LXR (140), or interfering with miRNA-regulated pathways (141), have been shown to elicit anti-inflammatory effects, due to both reduction of intracellular cholesterol concentrations but also altered intracellular cholesterol trafficking.

## 5 Conclusion

The cholesterol level of different organelle membranes ranges from approximately 30% to less than 1%. Intracellular vesicular and non-vesicular cholesterol trafficking mechanisms are important to maintain cholesterol homeostasis and distribution. Especially non-vesicular transport is likely to play a pivotal role in maintaining the cholesterol gradient across the secretory system from the ER via the Golgi network to the PM.

Taken together, the findings summarized and discussed in this mini review underline the important role of cholesterol trafficking in inflammatory processes. Even though it is widely accepted that changes in total cellular cholesterol levels as well as intracellular localization and trafficking can influence inflammatory pathway activation, detailed knowledge on their intertwining remains scarce. Further investigations are needed to decipher the fundamentals of non-vesicular cholesterol trafficking and the details of cholesterol transport at MCSs as well as the regulation of gene expression of cholesterol transporting proteins such as Aster proteins. Accordingly, a better understanding would help in the development of novel therapeutic targets and future pharmacological intervention strategies countering unbalanced cholesterol distribution and trafficking, especially in the context of inflammatory diseases.
